# Comparison of the Efficacy of S-1 Plus Oxaliplatin or Capecitabine Plus Oxaliplatin for Six and Eight Chemotherapy Cycles as Adjuvant Chemotherapy in Patients With Stage II-III Gastric Cancer After D2 Resection

**DOI:** 10.3389/fonc.2021.684627

**Published:** 2021-05-24

**Authors:** Yuanyuan Yu, Zicheng Zhang, Qianhao Meng, Yue Ma, Xiaona Fan, Jie Sun, Guangyu Wang

**Affiliations:** ^1^ Department of Gastrointestinal Medical Oncology, Harbin Medical University Cancer Hospital, Harbin, China; ^2^ School of Biomedical Engineering, School of Ophthalmology & Optometry and Eye Hospital, Wenzhou Medical University, Wenzhou, China

**Keywords:** adjuvant chemotherapy, gastric cancer, chemotherapy cycles, S-1 plus oxaliplatin, capecitabine plus oxaliplatin

## Abstract

**Objective:**

To compare the efficacy of adjuvant chemotherapy with six or eight cycles of S-1 plus oxaliplatin (SOX) or Capecitabine plus oxaliplatin (XELOX) after D2 resection of GC.

**Design and participants:**

We collected 470 cases of patients with TNM stage II and III GC who underwent D2 gastrectomy in the Harbin Medical University Cancer Hospital from January 2007 to December 2017 and received six or eight cycles of SOX or XELOX regimen. This study was designed to evaluate the prognosis of patients receiving six or eight cycles of SOX or XELOX chemotherapy and identify the appropriate number of chemotherapy cycles.

**Results:**

Among the 470 study participants [340 (72.3%) males; median age, 50 years (range, 24-76 years)], 355 and 115 received XELOX or SOX regimen chemotherapy, respectively. The number of 152 patients included in this study who received 6 and 8 cycles of chemotherapy in stage II and stage III without considering chemotherapy regimens were 125 and 27. The median DFS was, respectively, 14.9 months and 26.8 months (P = 0.08), the median OS was, respectively, 30.2 months and 30.8 months (P = 0.5), the difference was not statistically significant. Comprehensive survival analysis of XELOX and SOX group showed no significant difference for DFS (P = 0.29) and OS (P = 0.61). The total number of stage III GC patients who received six and eight cycles of chemotherapy was 92 and 19, respectively. The median DFS of patients who received six and eight cycles of chemotherapy was 14.6 and 23.2 months (P = 0.3), respectively. The median OS of patients who received six and eight cycles of chemotherapy was 26 and 30.6 months (P = 0.9), respectively. Comprehensive analysis of DFS (P=0.73) and OS (P=0.6) shows no difference between the XELOX group SOX groups. Subgroup analysis revealed significant differences in the gender (P = 0.05) and histological classification (P < 0.05) distribution.

**Conclusion:**

Regardless of the XELOX regimen or the SOX regimen, similar survival benefits are observed in patients receiving six or eight chemotherapy cycles irrespective of the regimen used. The XELOX and SOX regimens are well tolerated in patients undergoing D2 resection of GC.

## Introduction

Gastric cancer (GC) is the fifth most common cancer worldwide and the fourth leading cause of cancer-related death (1). GC disproportionally affects males, with the rate of affected males being almost twice that of affected females. In 2020, there will be an estimated 1.09 million new cases of GC worldwide and about 769,000 death (1). About 49.3% of new cancer cases and 58.3% of cancer deaths occur in Asia. In China, there were about 679,000 new cases of GC and 498,000 GC-related deaths in 2015, making makes GC the second to only lung cancer in terms of morbidity and mortality (2). In China, about 80% of patients with GC are at an advanced stage at the time of diagnosis, and the 5-year survival rate is less than 30% (3). Therefore, it is important to improve the prognosis of patients with stage II and stage III GC after surgery.

Postoperative adjuvant chemotherapy has become a routine treatment for patients with GC. Indications for adjuvant chemotherapy after resectable GC are: D2 gastrectomy and no preoperative treatment for postoperative patients with pathological stage II and III advanced GC. D2 gastrectomy is based on resectable GC. Four extensive clinical studies, the ACTS-GC, CLASSIC, JACCORGC-07 and ARTIST studies, have confirmed the value of postoperative adjuvant chemotherapy. The Japanese ACTS-GC trial confirmed that S-1 single-agent postoperative adjuvant chemotherapy could significantly improve the 5-year survival rate after D2 gastrectomy for locally advanced GC (4). However, this result has not been verified in other studies, and it is unclear whether patients with stage III GC can benefit from S-1 single-drug adjuvant chemotherapy. In response to this, the CLASSIC study, a randomized, open, parallel-controlled phase III clinical study involving patients from South Korea, China Mainland, and Taiwan, showed that GC patients who received XELOX adjuvant chemotherapy had a significantly higher 5-year disease-free survival (DFS) rate than did those who had surgery alone (68% vs. 53%; HR = 0.58; 95% CI: 0.47–0.72; P < 0.0001), and the overall survival (OS) rate was also significantly improved (78% vs. 69%; HR = 0.66; 95% CI: 0.51–0.85; P = 0.0015). These data confirm that XELOX regimen adjuvant chemotherapy can significantly reduce the risk of postoperative recurrence, and the benefits of prolonging DFS can then be translated into prolonging the OS of patients. As the first chemotherapy regimen validated by evidence-based medicine in the Chinese population, the classic study showed that the XELOX regimen is suitable for postoperative adjuvant chemotherapy for patients with stage II and II GC in China. It also further confirmed that postoperative adjuvant chemotherapy could play an essential role in treating locally advanced GC (5, 6). The Japanese JACCROGC-07 study is a randomized controlled study designed to evaluate the efficacy of S-1 combined with docetaxel in adjuvant treatment after surgery. The 3-year recurrence-free survival (RFS) of the S-1 combined with the docetaxel group was 7% higher than that of the control group. The 3-year RFS was significantly better in the treatment group than in the control group (65.9% vs. 49.6%, HR = 0.632, 99% CI: 0.400–0.998, P = 0.0007), and S-1 combined with docetaxel is recommended as the new standard for adjuvant treatment after D2 gastrectomy in patients with stage III GC (7). The Korean ARTIST study compared postoperative radiotherapy and chemotherapy after D2 surgery to postoperative adjuvant chemotherapy (Capecitabine combined with cisplatin). The results indicate that the DFS and OS of the two groups are similar. Subsequently, the ARTIST-II study, enrolling patients with GC and positive lymph nodes after D2, was designed. The results showed that compared with S-1 single-drug, the SOX regimen alone and in combination with radiotherapy can significantly prolong DFS. However, SOX regimen combined with radiotherapy did not improve survival when compared to SOX regimen alone (8–10). The results of these clinical studies indicate that surgery is the only possible cure for GC, and postoperative adjuvant chemotherapy is the main way to achieve long-term survival for patients with GC.

Based on the efficacy and safety of chemotherapy, the level I recommended choices for postoperative adjuvant chemotherapy for Chinese is XELOX and SOX. S-1 is a fluorouracil derivative, and the main components are tegafur, gemerazine, and otixiracet potassium. The curative effect of S-1 is equivalent to that of capecitabine. However, S-1 is superior to capecitabine in increasing the concentration and time of 5-FU in tumor tissue and blood and reducing side effects, including hand-foot syndrome (11, 12). In 2019, the RESOLVE study showed that eight cycles of SOX adjuvant chemotherapy after D2 radical resection of GC is not inferior to XELOX (13). Currently, eight chemotherapy cycles are recommended for patients with stage II and stage III GC, irrespective of whether they are undergoing the XELOX or SOX regimen. Individual differences in patients’ tolerance to chemotherapy drugs mean that some patients cannot tolerate the toxicity of chemotherapy drugs, leading to the early termination of chemotherapy. Therefore, for this group of patients, we aimed to compare the prognosis of patients who received six or eight cycles of XELOX or SOX adjuvant chemotherapy after radical resection of GC. These insights will allow practitioners to choose a suitable chemotherapy cycle for patients to avoid the occurrence of chemotherapy-related adverse reactions. Moreover, this data provides valuable evidence supporting the need for patients with advanced GC to receive standardized and individualized treatment, which can prolong their lives, improve their quality of life, and reduce the social burden on their families.

## Method

### Study Design

This is a retrospective study. In the real world, the proportion of people who can complete eight cycles of standard postoperative adjuvant chemotherapy for gastric cancer is not large. Most patients have completed six cycles of adjuvant chemotherapy. This research aims to compare whether six cycles are not inferior to eight cycles in the real world. We conducted a three-phase study. First, we compared the DFS and OS of patients with TNM stage II and III GC that received six or eight chemotherapy cycles without considering the chemotherapy regimen. Second, we compared the DFS and OS of patients who received six and eight chemotherapy cycles of XELOX and SOX. In the third step of the study design, the DFS and OS of patients with TNM stage III GC and 6 or 8 chemotherapy cycles were compared with and without considering the chemotherapy regimen.

### Inclusion Criteria

Data were collected from patients who were pathologically diagnosed with GC (GC)/gastric junction adenocarcinoma (GEJC) and had undergone D2 radical resection and postoperative adjuvant chemotherapy at the Harbin Medical University Cancer Hospital from January 2007 to December 2017. This study was approved by the Ethics Committee of the Harbin Medical University Cancer Hospital. Patient data were confidential, and the study complied with the Declaration of Helsinki.

Case inclusion criteria were (1): preoperative endoscopic biopsy or postoperative pathological diagnosis of GC/GEJC; (2) having undergone D2 radical operation; (3) postoperative pathological staging of stage II and stage III disease based on the American Joint Committee on Cancer (AJCC) TNM staging (8th edition); (4) postoperative hematology and imaging evaluation of patients show that they meet the criteria of postoperative adjuvant chemotherapy, and tolerate chemotherapy drugs; and (5) their postoperative adjuvant chemotherapy is XELOX or SOX.

Case exclusion criteria were: (1) patient received chemotherapy regimens other than S-1, SOX, or XELOX after D2 radical surgery for GC; (2) patient was unable to complete the specified adjuvant chemotherapy cycle as required for any reason; (3) for any reason, the standard chemotherapy dose was reduced by more than 30%; (4) patients received neoadjuvant therapy; and (5) patients with distant metastasis or relapse within 6 months during operation and after the operation, were excluded.

### Treatment Criteria

XELOX treatment regimen was: oxaliplatin 130 mg/m2 (intravenous drip) on day 1, repeated every three weeks and capecitabine 1000 mg/m2 (oral) on days 1-14, twice a day, repeated every three weeks. SOX treatment regimen was: oxaliplatin 130 mg/m2 (intravenous drip) on day 1, repeated every three weeks and S-1 40 mg/m2 (oral) on days 1-14, twice a day, repeated every three weeks. The two treatment groups were subject to 6 or 8 cycles of chemotherapy. During chemotherapy, symptomatic and supportive treatments including antiemetic, liver protection, and stomach protection were administered. B-ultrasound, CT and other imaging examinations were performed every three cycles to evaluate the treatment effect.

### Research Targets

DFS is defined as the time from the date of GC D2 resection to the occurrence of recurrence, metastasis, or death. The OS is the time from the date of GC D2 resection to death due to any cause. The primary study endpoint was 5-year OS, and the secondary study endpoint was 3-year DFS. All patients were followed up for at least five years.

### Statistical Methods

Clinicopathologic characteristics of patients receiving six and eight chemotherapy cycles were compared using the Chi-square test. The univariate Cox regression analysis to measure the association between treatment regimens and prognosis. The DFS and OS survival curves were drawn using the Kaplan-Meier method, and the log-rank test was used for comparison. P < 0.05 was considered statistically significant. All statistical analysis was performed with R Statistical Software (version 4.0.3).

## Result

### Patient Clinical Characteristics

The patient’s condition and tumor characteristics are shown in **Table 1**. This is a real-world study of patients with GC (stage II-III) who underwent D2 gastrectomy and received adjuvant chemotherapy with SOX or XELOX regimen in Harbin Medical University Cancer Hospital from January 2007 to December 2017. We conducted a retrospective study of 470 patients who underwent D2 resection and received postoperative adjuvant chemotherapy at our hospital and completed at least four cycles of adjuvant chemotherapy with either XELOX or SOX regimens. There were 159 patients available for analysis of DFS and 203 patients available for analysis of OS. Postoperative pathological staging was stage II or stage III in 369 patients, of which 290 patients received six cycles of chemotherapy and 79 patients received eight cycles of chemotherapy. Following the administration of chemotherapy, 51 patients were excluded based on the exclusion criteria. A total of 152 patients were included in the analysis according to the exclusion criteria.

**Table 1:** Clinical characteristics of patients with gastric cancer after D2 resection enrolled in this study.

Clinical characteristics	Cycle 6 (n=290)	Cycle 8 (n=79)	X-squared	p-value^a^
Age				
<=65	248	60	3.455	0.063
>65	42	19		
Gender				
Male	196	63	3.826	0.05
Female	94	16		
Lauren				
Intestinal type	60	14	1.678	0.642
Diffuse type	73	24		
Mixed type	43	14		
Unknown	114	27		
Tumor_size				
Cardia	9	2	1.540	0.463
Gastric body or Whole stomach	82	28		
Gastric antrum	199	49		
TNM				
II	127	34	<0.001	1
III	163	45		
WHO_grade				
Adenocarcinoma	155	42	0.111	0.991
Signet ring cell carcinoma	19	5		
Low adhesion carcinoma	19	6		
Mixed cancer	97	26		
Histological classification				
Poorly differentiated	142	27	7.914	0.048
Moderately differentiated	124	47		
Well differentiated	10	1		
Undifferentiated	14	5		

^a^p-value was derived from the Chi-square test.

The clinical characteristics of the 6-cycle and 8-cycle chemotherapy groups were similar. There were no significant differences in Lauren classification, tumor location, TNM staging, or WHO grade (P > 0.05). There was an obvious difference between these two groups in age distribution, with most patients under 65 years of age, but this difference was not statistically significant (P=0.063). Obvious differences in gender distribution (P = 0.05) and histological classification (P = 0.48) were observed (**Table 1**).

### Survival Outcome for Stage II-III GC Patients Treated With Six or Eight Chemotherapy Cycles

**Figure 1:**
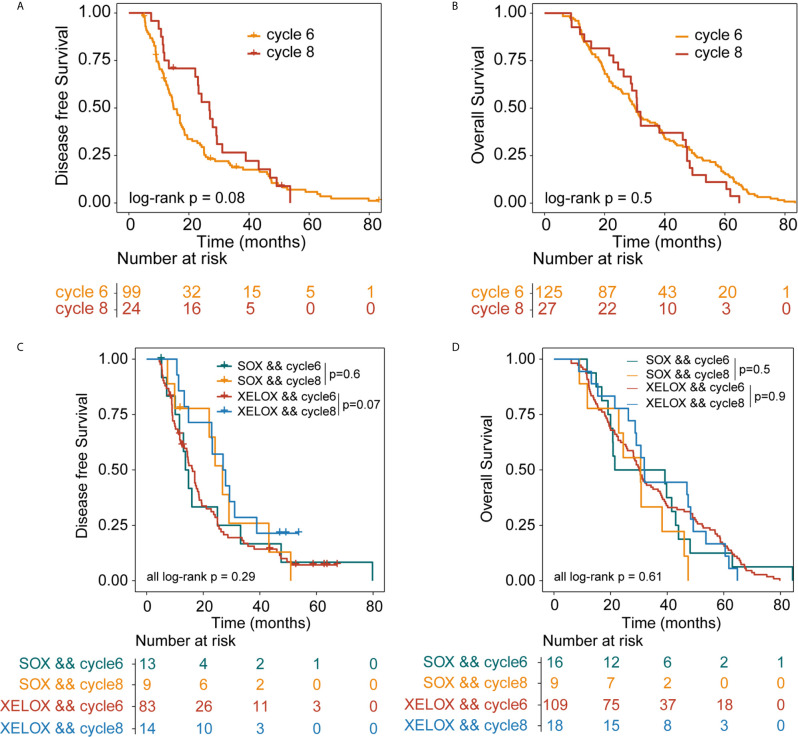
Kaplan-Meier survival curves for disease-free survival (DFS) and overall survival (OS). DFS **(A)** and OS **(B)** analyses for stage II and stage III patients who received six or eight cycles of chemotherapy, irrespective of whether they underwent XELOX or SOX regimens. DFS **(C)** and OS **(D)** analyses for stage II and stage III patients who received six or eight cycles of chemotherapy, taking the specific regimen into account. XELOX, Capecitabine plus oxaliplatin; SOX, S-1 plus oxaliplatin.

**Figure 2:**
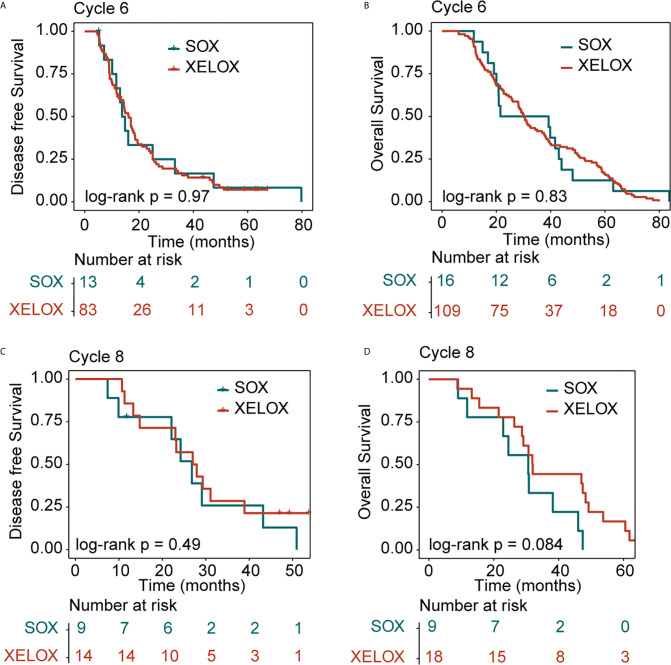
Kaplan-Meier survival curves for disease-free survival (DFS) and overall survival (OS). DFS **(A)** and OS **(B)** analyses for the XELOX and SOX regimens for patients with stage II and stage III gastric cancer receiving six cycles of chemotherapy. DFS **(C)** and OS **(D)** analyses for the XELOX and SOX regimens for patients with stage II and stage III gastric cancer receiving eight cycles of chemotherapy. XELOX, Capecitabine plus oxaliplatin; SOX, S-1 plus oxaliplatin.

All patients were followed up for at least 5 years. We conducted a comprehensive analysis of the data for patients with stage II and stage III, irrespective of whether they underwent XELOX or SOX regimens. Patient survival was then compared between groups. The number of patients receiving six and eight cycles of chemotherapy was 125 and 27, respectively. Patients receiving six or eight chemotherapy cycles had similar rates of DFS and OS. The Kaplan–Meier method was used for survival analysis and to draw DFS and OS survival curves. Median DFS time of patients receiving six and eight cycles of chemotherapy was 14.9 and 26.8 months (P = 0.08), respectively. Median OS time of patients receiving six and eight cycles of chemotherapy was 30.2 and 30.8 months (P = 0.5) (**Figures 1A, B**). The number of patients with stage II and stage III GC receiving six and eight cycles of XELOX regimen chemotherapy were 109 and 18, respectively. Median DFS time of patients receiving six and eight cycles of chemotherapy was 16 and 27 months (P = 0.07), respectively. The median OS times of patients receiving six and eight cycles of chemotherapy, respectively, were 30 and 31.9 months (P = 0.9. The number of patients receiving six and eight cycles of SOX regimen chemotherapy was 16 and 9, respectively. The median DFS times in these patient groups were 13.7 and 24.2 months (P = 0.6), respectively. The median OS times in these patient groups were 21.5 and 24.5 months (P = 0.5), respectively. Comprehensive analysis of DFS (P = 0.29) and OS (P = 0.61) between the XELOX and SOX groups revealed no statistical difference (**Figures 1C, D**). In patients receiving six chemotherapy cycles, DFS and OS did not differ between those receiving XELOX and SOX regimens (DFS, P = 0.97 and OS, P = 0.83) (**Figures 2A, B**). In patients receiving eight chemotherapy cycles, DFS and OS did not differ between those receiving the XELOX and the SOX regimens (DFS, P = 0.49 and OS, P = 0.084) (**Figures 2C, D**).

**Figure 3:**
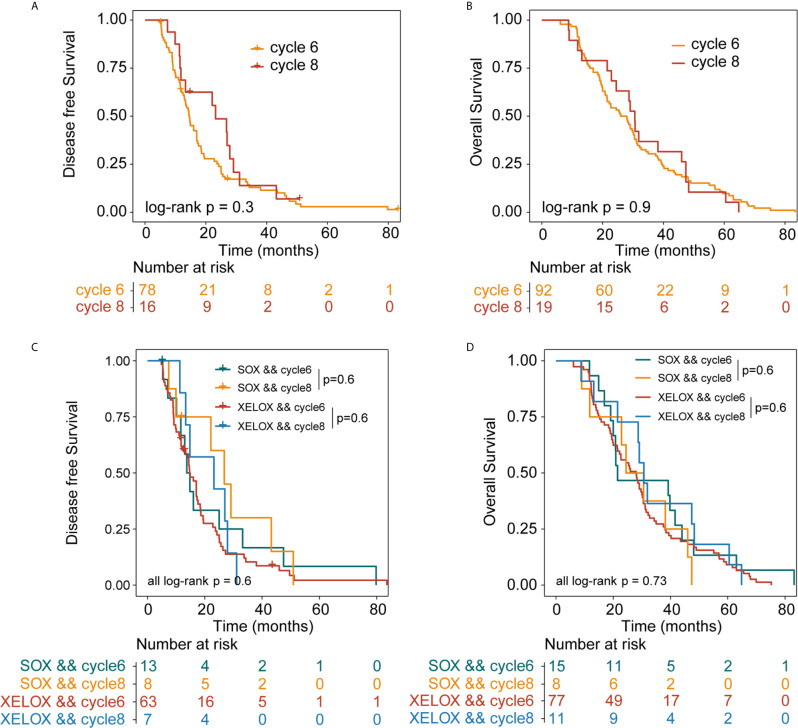
Kaplan-Meier survival curves for disease-free survival (DFS) and overall survival (OS). DFS **(A)** and OS **(B)** analyses for patients with stage III gastric cancer who received six or eight cycles of chemotherapy, irrespective of whether they underwent XELOX or SOX regimens. DFS **(C)** and OS **(D)** analyses for patients with stage III gastric cancer who received six or eight cycles of chemotherapy, taking the specific regimen into account. XELOX, Capecitabine plus oxaliplatin; SOX, S-1 plus oxaliplatin.

Among all patients with stage III GC, 92 and 19 received six and eight chemotherapy cycles, respectively. Overall DFS time in patients receiving six and eight cycles of chemotherapy were 14.6 and 23.2 months (P = 0.3), respectively. The median OS times in patients receiving six and eight cycles of chemotherapy were 26 and 30.6 months (P=0.9), respectively (**Figures 3A, B**).

**Figure 4:**
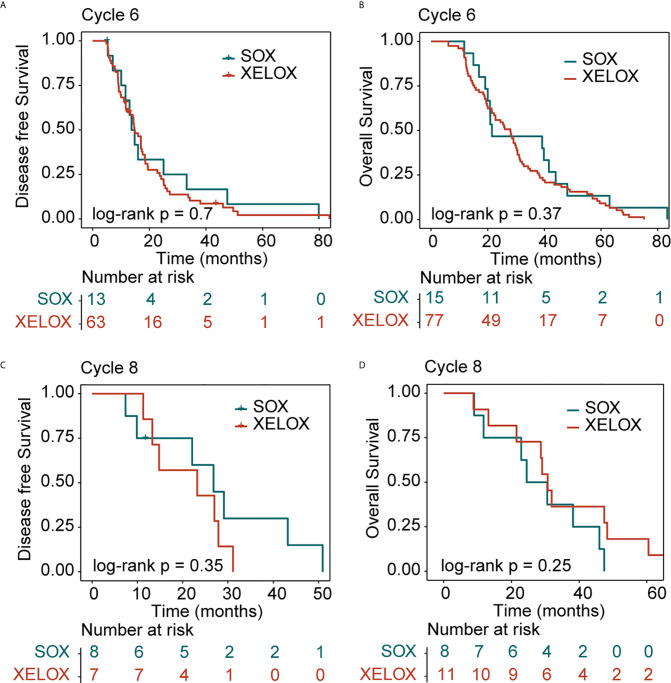
Kaplan-Meier survival curves for disease-free survival (DFS) and overall survival (OS). DFS **(A)** and OS **(B)** analyses for XELOX and SOX regimens for patients with stage III gastric cancer receiving six cycles of chemotherapy. DFS **(C)** and OS **(D)** analyses for the XELOX and SOX regimens in stage III patients receiving eight cycles of chemotherapy. XELOX, Capecitabine plus oxaliplatin; SOX, S-1 plus oxaliplatin.

In patients with stage III GC, 77 and 11 patients received six and eight cycles of the XELOX regimen chemotherapy, respectively. The median DFS times for patients receiving six and eight cycles of chemotherapy were 14.7 and 23.2 months (P = 0.6), respectively. The median OS times for patients receiving six and eight cycles of chemotherapy, 28 and 30.7 months (P = 0.6), respectively. the difference was not statistically significant. The number of people receiving six and eight cycles of SOX regimen chemotherapy was 15 and 8, respectively. The median DFS times for patients receiving six and eight cycles of chemotherapy were 13.7 and 26.8 months (P = 0.6), respectively. The median OS times for patients receiving six and eight cycles of chemotherapy were 21.5 and 24.5 months (P= 0.6), respectively. No differences in DFS (P = 0.73) and OS (P=0.6) were observed between the XELOX and SOX groups (**Figures 3C, D**). In patients receiving six chemotherapy cycles, DFS and OS did not significantly differ between the two treatment regimens (DFS, P = 0.7 and OS, P = 0.37) (**Figures 4A, B**). In patients receiving eight chemotherapy cycles, DSF and OS did not differ between the two treatment groups (DFS, P = 0.35 and OS, P = 0.25) (**Figures 4C, D**).

**Figure 5:**
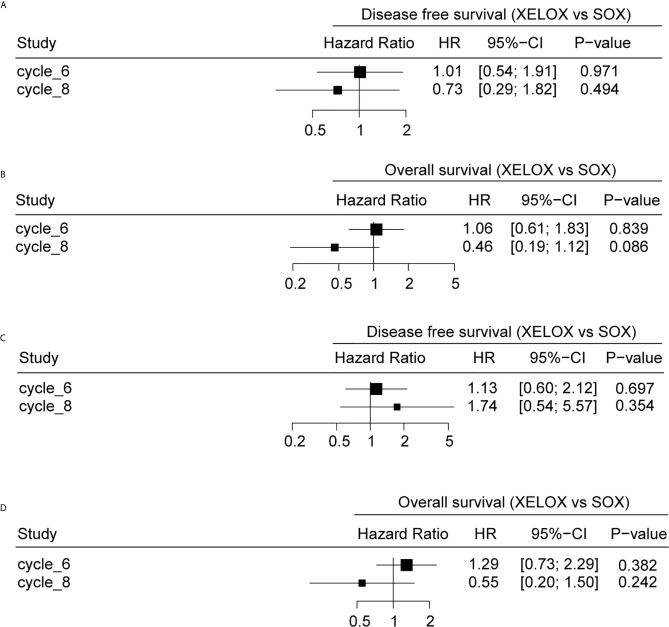
The relationship between different chemotherapy cycles and disease-free survival (DFS) and overall survival (OS) of gastric cancer patients. DFS **(A)** and OS **(B)** analyses for patients with stage II and stage III gastric cancer. DFS **(C)** and OS **(D)** analyses for stage patients with stage III gastric cancer.

Results of the univariate Cox regression suggested that there is no difference in survival between patients receiving six or eight chemotherapy cycles in either of the treatment regimens examined (P>0.05) (**Figures 5A–D**). Patients receiving eight cycles of XELOX regimen chemotherapy appeared to have better OS than did those receiving eight cycles of SOX or XELOX regimen chemotherapy, but this difference was not statistically significant (OS: HR, 0.46; P = 0.086) (**Figure 5B**).

### Subgroup Analysis

**Figure 6:**
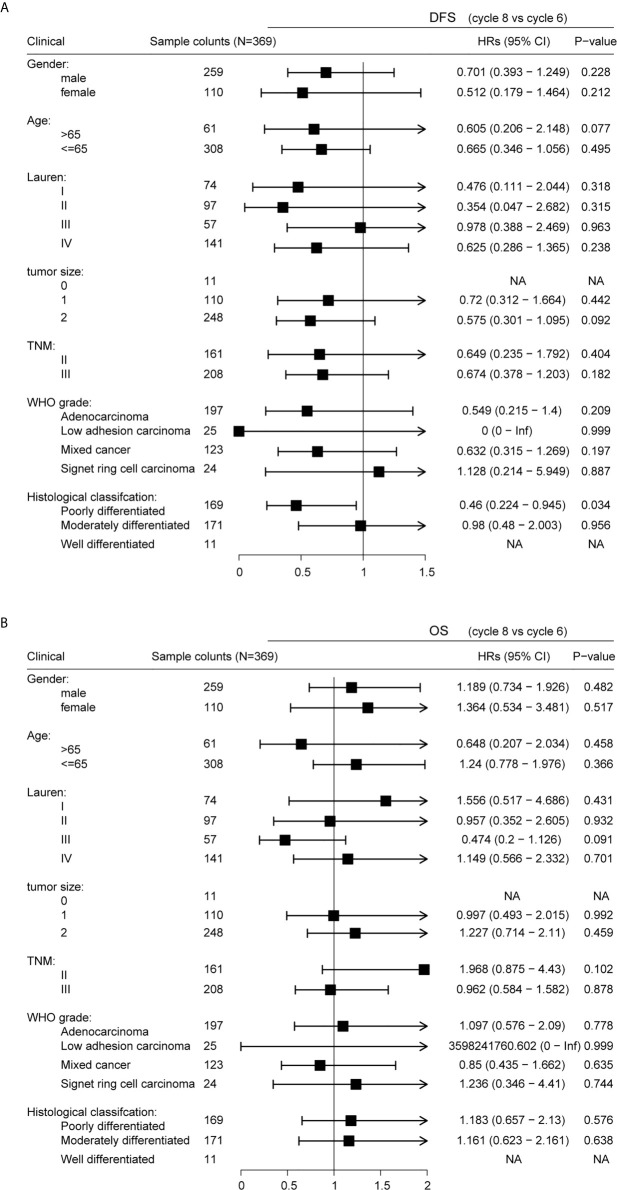
Subgroup analyses of disease-free survival (DFS) **(A)** and of overall survival (OS) **(B)**.

Stratification by gender, age, Lauren classification, tumor location, TNM staging, WHO grade, and histological classification revealed similar DFS results for patients receiving six and eight cycles of chemotherapy (**Figure 6A**; P > 0.05). However, a significant difference was observed in DFS in patients classified as poorly differentiated histologically (P = 0.034), suggesting that six cycles of chemotherapy for patients with GC histologically classified as poorly differentiated should be sufficient. Stratification by gender, age, Lauren classification, tumor location, TNM staging, WHO grade, and histological classification revealed similar that OS for all patients irrespective of whether they received six or eight cycles of chemotherapy (**Figure 6B**; P>0.05).

## Discussion

GC is a highly invasive and highly heterogeneous disease. The survival rate of locally advanced or metastatic disease of GC has not been significantly improved. It is still a serious global health problem. GC passes through the lymphatic system, blood and peritoneum in the early stage. Spread, recurrence after surgery is common, about 40% of patients relapse within 2 years after surgery (14–16). In order to reduce the rate of local recurrence and metastasis of GC and prolong the survival time of patients, we routinely perform postoperative adjuvant chemotherapy for patients. SOX regimen and XELOX regimen are the first-line treatment options for advanced GC, reducing cancer recurrence, improving the survival rate, and reducing the occurrence of adverse reactions, so that the survival of patients with advanced GC has obvious benefits (17–21). In recent years, a number of large randomized clinical studies have also confirmed the status of the two regimens in adjuvant chemotherapy after GC surgery. The purpose of our research is to compare the efficacy of patients receiving 6, and 8 cycles of SOX and XELOX adjuvant chemotherapy after D2 radical resection of GC, and to compare the prognosis of patients receiving different chemotherapy cycles. As far as we know, this idea was proposed for the first time. Regardless of how many cycles of chemotherapy the patient received, we did not observe significant differences between the two regimens in DFS and OS. In all subgroup analyses, only the distribution of patients classified as poorly differentiated histologically in 6 and 8 chemotherapy cycles was significantly different (P=0.034).

Previous prospective studies on the adjuvant treatment of GC, the ACTS-GC, CLASSIC, and ARTIST II studies, showed that compared with surgery alone, S-1, SOX and XELOX regimens have better curative effects, but these studies did not directly compare the efficacy of SOX regimen and XELOX regimen. Therefore, the difference in the efficacy of these two regimens was still unknown at that time. A single-center retrospective study showed that there was no significant difference in the efficacy of S-1 and XELOX regimens in stage III patients, but XELOX regimen was more effective than S-1 in patients with stage IIIC GC (22). Another multi-center retrospective study showed that for patients with stage IIIB or IIIC GC after D2 lymph node dissection, XELOX regimen adjuvant chemotherapy is more effective than S-1 (23). For the comparison of the effects of SOX and XELOX, a Japanese study showed that XELOX and SOX treatments have similar effects in patients with stage III GC who underwent D2 resection (24). Subsequently, the RESOLVE study published by ESMO in 2019 showed that the SOX adjuvant chemotherapy for 8 cycles after radical resection of GC D2 is not inferior to XELOX (25). The results of a recent single-center retrospective study also showed that SOX is as effective as XELOX for patients with GC after radical resection and that there is no significant difference in survival rate in patients receiving the different treatments (13).

The limitations of this study should be taken into consideration when analyzing the results. First, this is a single-center retrospective study, and the data collected will inevitably have some deviations. Second, the number of patients included is small and the sample distribution is uneven. These factors may affect the experimental results. Therefore, to verify the accuracy of our results, it is necessary to conduct large-scale prospective clinical randomized controlled trials.

Together, these results and ours presented here show that the SOX chemotherapy regimen is not inferior to the XELOX regimen. Therefore, it is appropriate to compare the survival and prognosis of patients with GC receiving six and eight chemotherapy cycles, irrespective of whether they underwent SOX or XELOX regimens. Our results suggest that for patients with stage III GC, eight cycles of chemotherapy are not more effective than six cycles with regards to DFS and OS. We propose that clinically, for patients with stage III GC, six chemotherapy cycles are effective and decrease the occurrence of chemotherapy-related adverse reactions. This result needs to be verified, but may help patients with GC choose the number of adjuvant chemotherapy cycles after surgery, avoid unnecessary increased rounds of chemotherapy, improve the quality of life, and reduce family burdens.

## Conclusion

Six chemotherapy cycles of SOX or XELOX are as effective as eight cycles in patients with TNM stage III GC after D2 radical resection.

## Data Availability Statement

The original contributions presented in the study are included in the article/supplementary material. Further inquiries can be directed to the corresponding authors.

## Author Contributions

GW and JS conceived the study. YY, ZZ and QM performed data analysis and wrote the manuscript. XF and YM provided technical guidance. YY and QM carried out data collection. GW finalized the research results and the final version of the manuscript. All authors contributed to the article and approved the submitted version.

## Funding

This work was supported by grants from Haiyan Fund Key Project (JJZD2018-05).

## Conflict of Interest

The authors declare that the research was conducted in the absence of any commercial or financial relationships that could be construed as a potential conflict of interest.
